# Histological and mast cells evaluation after application of botulinum toxin type a in masseter muscles of rats

**DOI:** 10.4317/jced.59960

**Published:** 2022-12-01

**Authors:** Rodrigo Blas, Ana-Amelia Souza, Regiane Silva, Marcelo-Henrique Napimoga, Juliana-Trindade-Clemente Napimoga, Vera-Cavalcanti de Araújo, Andresa-Borges Soares

**Affiliations:** 1 Department of Oral Pathology, São Leopoldo Mandic Institute and Research Center, Campinas, SP, Brazil; 2 Faculdade São Leopoldo Mandic, Institute and Research Center, Laboratory of Neuroimmune Interface of Pain Research, Campinas, SP, Brasil

## Abstract

**Background:**

Botulinum Toxin Type A (BTX-A) has been largely used to reduce muscle strength of masseter and temporal muscles by producing a temporary weakening of their activity. This study aimed to evaluate the histological changes and the number of mast cells after the injection of BTX-A.

**Material and Methods:**

In the masseter muscle of rats in the periods of 1, 7, 15, and 30 days. These muscles were stained with hematoxylin and eosin (H&E) and toluidine blue (TBO). The presence or absence of an inflammatory process and necrosis were analyzed by H&E in all area of the slide at 10X magnification. The number of mast cells was evaluated by counting 10 “hotspots” in the intra-muscular region on TBO-stained slides, 400X magnification. Statistical analysis was performed through two-way analysis of variance and Tukey’s test.

**Results:**

As a result, the inflammatory process and necrosis were not observed in any periods studied in both groups Regarding mast cells, there was no statistically significant increase in their quantity in the study group when compared to the control group in the evaluation periods of 7 days and 15 days. However, these mast cells increased significantly during the periods of 1 and 30 days.

**Conclusions:**

This study showed that even in the absence of an inflammatory process, there was an increase in the number of mast cells in the first 24 hours after the application of BTX-A, with a subsequent balance between the numbers of mast cells at 7 and 15 days, and again an increase after 30 days.

** Key words:**Botulinum toxins type A, mast cells, masseter muscle.

## Introduction

Mast cells are inflammatory cells originated of bone marrow that are part of innate and acquired immunity with the immediate and late release of various chemical mediators. When activated, these cells synthesize and release important immunoregulatory cytokines mobilizing a fast and vigorous inflammatory response (1-3).

Since mast cells collaborate with immune reactions and have a fundamental role in the inflammatory response, the aim of this study is histological and mast cell evaluation after the action of BTX-A, Botox®, in the periods of 1, 7, 15, and 30 days.

## Material and Methods

This study was approved by the animal ethics committee of São Leopoldo Mandic number 2018/006.

Twenty Rattus norvegicus albinus, Wistar lineage were selected. The animals were kept under controlled conditions. The animals were divided into four groups with five animals in each group: group I - 24 hours, group II - 7 days, group III - 15 days, and group IV - 30 days. All animals received 1 µl (corresponding to 1U) of BTX-A (Botox®) in the right masseter muscle (study group) and 1 µl of 0.9% sterile saline solution in the left masseter muscle (control group), under general anesthesia.

The rats were euthanized by deepening anesthesia after the periods of 24 hours, 7 days, 15 days, and 30 days. The masseter muscle was then removed, and the resulting fragments were immersed in a 10% formalin solution for 24 hours.

The paraffin-embedded tissues were cut following a 4 µm-thick pattern for the preparation of H&E-stained (hematoxylin and eosin) slides, which were all analyzed by a pathologist for the presence or absence of an inflammatory process and necrosis.

A histochemical reaction of Toluidine blue was used to identify mast cells. First, the slide was analyzed in its entire length, and subsequently, ten “hotspots” intramuscular areas were chosen for the mast cell count in a magnification of 400X.

Statistical analysis was carried out through a two-way analysis of variance for randomized blocks and Tukey’s test. Statistical calculations were conducted by using the SPSS 23 (SPSS Inc., Chicago, IL, USA), considering a 5% significance level.

## Results

Histologically, no inflammatory process was observed, both in the study group and in the control group in none of the studied times (1, 7, 15, and 30 days).

The two-way analysis of variance for randomized blocks demonstrated that the number of mast cells in the masseter muscle was significantly affected by the interaction between BTX-A application and time (*p* = 0.033).

**Figure 1:**
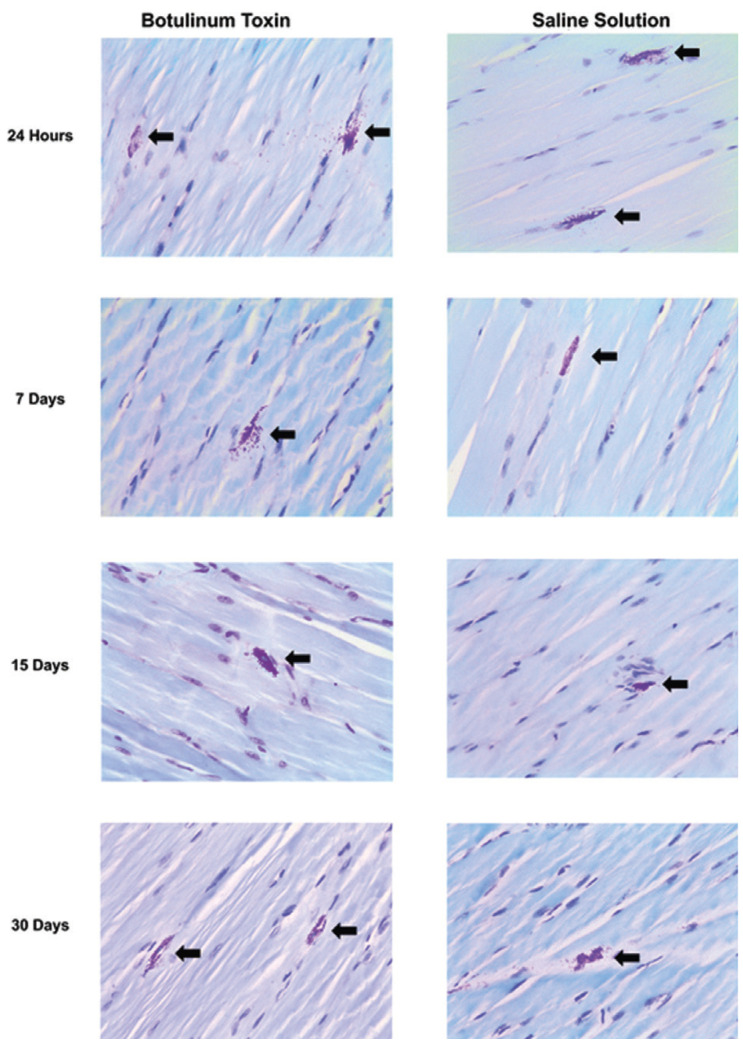
Presence of mast cells in the masseter muscle of rats stained in toluidine blue at a 400X magnification in the Botulinum Toxin group and in the Saline Solution group at 24 hours, 7 days, 15 days, and 30 days.

**Figure 2:**
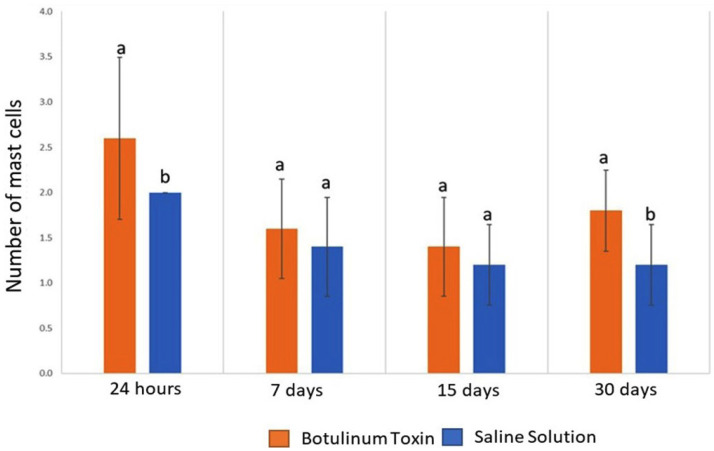
Bar chart of the number of mastocytes in the masseter muscle, over time, after application of the botulinum toxin type A.

After 24 hours, the number of mast cells in the masseter muscle following the application of BTX-A significantly increased when compared to the saline solution. After 7 and 15 days, there was no significant statistical difference in mast cell count when comparing BTX-A application and saline solution application. At 30 days, the number of mast cells was significantly higher in the study group compared to the control group (Figs. 1,2).

## Discussion

This study showed that intramuscularly injected BTX-A did not present any histological alteration in any of the times studied. However, it showed a surprising increase in the number of mast cells in groups 24hours and 30 days after application.

Although this study did not show histological alterations, an interesting finding was a statistically significant increase in mast cells in specimens treated with BTX-A in the periods of 24hours and 30 days. In this study, the presence of degranulated mast cells was observed both in the control and study groups. However, the presence of inflammatory cells was not identified in any of the studied times, suggesting that this increase in the number of mast cells in the group treated with BTX-A is possibly not related to its role in inflammatory processes.

The application of BTX-A has been related to antipruritic effects in several disorders such as scratching rash (1-4), and lichen (5). This action of BTX-A is believed to be due to inhibition of the mast cells’ secretory granules (6). These results differ from our findings. However, it is important to mention that these studies were carried out with subcutaneous injections, differently from what is recommended in the study, in which BTX-A was administered intramuscularly.

Park (7) presented the only study analyzing mast cells histologically and demonstrated a decrease in the number of mast cells after 7 days of BTX-A application to skin tissue along with muscle. In disagreement with the findings by Park (7), this study observed a statistically significant increase in the period of 24 hours and 30 days. Although there was no statistical difference, an increase in mast cells was also identified at 7 and 14 days. A possible explanation for this difference in results may be related to the fact that BTX-A was applied directly to the muscle in the present study, whereas in Park’s article (7), it was applied to the skin and muscle and it is likely that the analysis of mast cells has been performed in the connective tissue area, whereas in this study the analysis was performed only in intramuscular areas. Another reason for this difference can be attributed to the fact that the current research focused on the masseter muscle, an extremely active muscle in rodents.

## Conclusions

Histologically, there was no inflammatory process or necrosis of the masseter muscle after BTX-A injection in rats, with no difference between groups. With respect to mast cells, a statistically significant increase in their intramuscular numbers was observed in the periods of immediate (24hours) and late (30days) in the BTX-A-treated group when compared to the control group.
